# A RE-AIM evaluation of evidence-based multi-level interventions to improve obesity-related behaviours in adults: a systematic review (the SPOTLIGHT project)

**DOI:** 10.1186/s12966-014-0147-3

**Published:** 2014-12-06

**Authors:** Sofie Compernolle, Katrien De Cocker, Jeroen Lakerveld, Joreintje D Mackenbach, Giel Nijpels, Jean-Michel Oppert, Harry Rutter, Pedro J Teixeira, Greet Cardon, Ilse De Bourdeaudhuij

**Affiliations:** Department of Movement and Sport Sciences, Faculty of Medicine and Health Sciences, Ghent University, Ghent, Belgium; Research Foundation Flanders (FWO), B-1000 Ghent, Belgium; The EMGO Institute for Health and Care Research, Department of Epidemiology and Biostatistics, VU University Medical Center, Amsterdam, The Netherlands; The EMGO Institute for Health and Care Research, Department of General Practice and Elderly Care Medicine, VU University Medical Center, Amsterdam, The Netherlands; Université Paris 13, Sorbonne Paris Cité - UREN (Unité de Recherche en Epidémiologie Nutritionnelle), U557 Inserm; U1125 Inra; Cnam, Centre for Research on Human Nutrition Ile-de-France (CRNH IdF), Bobigny, France; Université Pierre et Marie Curie-Paris 6, Department of Nutrition Pitié-Salpêtrière Hospital (AP-HP), (CRNH IdF), Institute of Cardiometabolism and Nutrition (ICAN), Paris, France; European Centre on Health of Societies in Transition, London School of Hygiene and Tropical Medicine, London, UK; Interdisciplinary Center for the Study of Human Performance, Faculty of Human Kinetics, University of Lisbon, Lisbon, Portugal

**Keywords:** RE-AIM, Overweight, Obesity, Nutrition and physical activity interventions, Review

## Abstract

**Background:**

This systematic literature review describes the potential public health impact of evidence-based multi-level interventions to improve obesity-related behaviours in adults, using the Reach, Efficacy, Adoption, Implementation and Maintenance (RE-AIM) framework.

**Methods:**

Electronic databases (PubMed, Embase, and The Cochrane Library) were searched to identify intervention studies published between January 2000 and October 2013. The following inclusion criteria were used: (1) the study included at least one outcome measure assessing obesity-related behaviours (i.e. diet, physical activity or sedentary behaviour), (2) the study collected data over at least one year and (3) the study’s intervention targeted adults, was conducted in a specified geographical area or worksite, and was multi-level (i.e. targeting both individual and environmental level). Evidence of RE-AIM of the selected interventions was assessed. Potential public health impact of an intervention was evaluated if information was provided on at least four of the five RE-AIM dimensions.

**Results:**

Thirty-five multi-level interventions met the inclusion criteria. RE-AIM evaluation revealed that the included interventions generally had the potential to: reach a large number of people (on average 58% of the target population was aware of the intervention); achieve the assumed goals (89% found positive outcomes); be broadly adopted (the proportion of intervention deliverers varied from 9% to 92%) and be sustained (sixteen interventions were maintained). The highest potential public health impact was found in multi-level interventions that: 1) focused on all levels at the beginning of the planning process, 2) guided the implementation process using diffusion theory, and 3) used a website to disseminate the intervention.

**Conclusions:**

Although most studies underreported results within the RE-AIM dimensions, the reported Reach, Effectiveness, Adoption, Implementation and Maintenance were positively evaluated. However, more information on external validity and sustainability is needed in order to take informed decisions on the choice of interventions that should be implemented in real-world settings to accomplish long-term changes in obesity-related behaviours.

**Electronic supplementary material:**

The online version of this article (doi:10.1186/s12966-014-0147-3) contains supplementary material, which is available to authorized users.

## Background

The growing prevalence of overweight (Body Mass Index ≥25 kg/m^2^) and obesity (Body Mass Index ≥30 kg/m^2^) in adults is a major public health concern in European countries. Overweight and obesity contribute to mortality and the burden of many chronic diseases, such as cardiovascular diseases, cancer, type 2 diabetes and osteoarthritis [1-4]. Depending on country and gender, the overall prevalence of overweight in Europe currently ranges from 39.3% (France) to 64.9% (England) in men and from 21.9% (Italy) to 51.4% (England) in women. The overall prevalence of obesity ranges from 6.0% (France) to 21.6% (England) in men and from 5.0% (Italy) to 23.3% (England) in women [5]. In several European countries, adult obesity rates have doubled during the last two decades [6-8]. Given the serious health consequences and the rapidly increased prevalence, the development and implementation of effective, sustainable overweight and obesity prevention approaches is imperative.

In the past, several theoretical models have been used to develop overweight and obesity prevention approaches. Many of these approaches were informed by social psychological theories such as the Theory of Planned Behaviour [9], and the Transtheoretical Model [10], and were thus focused on health education and the modification of individual-level determinants of obesity-related behaviours (i.e. dietary, physical activity and sedentary behaviours) [11,12]. Athough these individual-based interventions have sometimes shown short-term effects, their long-term effectiveness is generally limited [12-14]. This could be explained by the fact that health behaviours are not solely a matter of individual determinants, but are also strongly affected by environmental factors [15-17]. Environmental factors are of growing importance with the development of increasingly ‘obesogenic’ environments in recent decades characterized by readily available, cheap, heavily advertised energy-dense foods, often provided with large portion sizes, and by reduced opportunities for physical activity accompanied by increased likelihood of sedentary behaviour, due to features such as urban sprawl, a lack of perceived safety and reductions in walkability [17-20]. These obesity-related environmental factors can be categorized using the Analysis Grid for Environments Linked to Obesity (ANGELO) framework [21]. This framework consist of two axes representing the size (micro vs. macro) and the type of the environment (i.e. socio-cultural, economic, political or physical environmental level).

Consequently, multi-level interventions that target both the individual level (e.g. by changing the beliefs, attitudes and knowledge of the participants) and at least one of the environmental levels as defined by the Analysis Grid for Environments Linked to Obesity (ANGELO) framework (i.e. socio-cultural, economic, political or physical environmental level) have shown promising results in counteracting obesity [11,14,22-24].

However, despite the increasing interest in multi-level interventions, little information is available on their characteristics, effectiveness and external validity [25]. Information on generalizability is essential to translate research findings into practice. To gain insight into both internal and external factors of health promotion interventions, Glasgow and colleagues developed the RE-AIM framework. This framework focuses on the five most important dimensions for evaluating the potential public health impact of programs intended for wide-scale implementation and dissemination. The framework covers the degree to which (1) an intervention reaches the target population, and to which degree the intervention participants are representative of the non-participants; (2) an intervention achieved the assumed goals, with optimal quality of life and without negative outcomes; (3) an intervention was broadly adopted, and to which degree both delivery setting and delivery staff were representative of non-deliverers; (4) an intervention was consistently implemented at a reasonable cost; and (5) an intervention had the ability to be sustained, with long-lasting individual effects [26].

The aim of the present study was to conduct a systematic review of multi-level interventions, aimed at reducing obesity-related behaviours in adults, as part of the European Commission funded “sustainable prevention of obesity through integrated strategies” (SPOTLIGHT) project [27]. The purpose of this review was to gain insight into 1) the characteristics of multi-level intervention, 2) the internal and external validity factors of multi-level interventions, and 3) the potential public health impact of multi-level interventions.

## Methods

### Literature search

A systematic literature search of three electronic databases (PubMed, Embase, The Cochrane Library) was conducted in April 2012, and updated in October 2013 to detect relevant intervention studies. The search strategy was developed using the PICO (participant, intervention, comparison, outcome) approach, and was limited to the English literature, published between January 2000 and October 2013. Details on the search strategy are listed in Additional file 1: Table S1.

After running the search strategy, duplicates were identified and removed. Subsequently, the studies were screened by title, abstract and full text to determine their eligibility by one reviewer (SC) and independently checked by a second reviewer (KDC). In addition, reference lists from the retrieved articles were examined for additional relevant intervention studies.

### Inclusion criteria

To be eligible, intervention studies had to meet the following inclusion criteria: (1) the study included at least one outcome measure assessing obesity-related behaviours (i.e. dietary, physical activity and sedentary behaviour); (2) the study collected data over at least one year; and (3) the study intervention was community-based, multi-level, and targeted adults. Interventions were considered community-based if they targeted a group of people that were mutually connected by the geographical area in which they were living or the worksite in which they were working. Interventions were considered multi-level, if they targeted at least one individual-level and at least one environmental-level determinant of obesity-related behaviour. Environmental-level determinants were classified into four types based on the Analysis Grid for Environments Linked to Obesity (ANGELO) framework: physical environmental factors, socio-cultural environmental factors, economic environmental factors and political environmental factors.

### Data extraction

As a result of the screening process, 35 interventions were selected. These are listed in Additional file 2, which presents information on the focus of the intervention (i.e. physical activity, sedentary behaviour or eating behaviour), study design, target population, study participants, intervention components, outcome measures and geographical area. Study design was divided into the categories of pre-experimental (one group pre-test post-test design and one group post-test only design) and experimental studies ((cluster) randomized controlled trials). As only multi-level interventions were included, the intervention components were split up into individual and environmental level components. Individual components aimed to change psychological factors, such as beliefs or knowledge (e.g. via information sessions, posters, etc.), while environmental components target the sociocultural (e.g. walking groups), economic (e.g. reduction of prices of healthy food items), political (e.g. the earning of physical activity points, which could be redeemed for paid leave) or physical environment of the participants (e.g. provision of cycling infrastructure). Outcome measures were considered in the categories of overweight and obesity-related behavioural outcomes (dietary behaviour, physical activity and sedentary behaviour) and obesity-related physiological outcomes (e.g. BMI, weight, fat percentage).

### RE-AIM evaluation

The included interventions were evaluated on the basis of the RE-AIM framework [26]. Each of the five RE-AIM dimensions was divided into a number of indicators, and all included articles were coded by the first author on whether they reported on these specific indicators. A random selection of one third of the interventions was also coded by the second author to determine inter-rater reliability (ICC = 0.81). Differences were discussed between the two assessors until full consensus was reached. The indicators for reach were: the description of the target population, awareness/participation rate, characteristics of people aware of the intervention and their representativeness. For effectiveness, we coded whether a study reported on positive outcomes, quality of life, negative outcomes and short-term attrition. Adoption was coded based on the following indicators: the proportion and representativeness of staff who delivered the intervention within the intervention delivery settings, and the proportion and representativeness of intervention delivery settings and non-delivery settings. Implementation was coded on completeness and fidelity of implementation and time, financial investment and staff expertise needed to implement the intervention. Maintenance was split up into individual level and organizational level maintenance. Individual level maintenance was based on whether information was reported on long term effectiveness, i.e. were outcomes reported at least six months after the completion of the intervention study: six months is a widely used time frame to assess behaviour change maintenance [28,29]. Organizational level maintenance was based on program sustainability, program adaptations and representativeness of settings/agents who were still delivering the interventions after the intervention study had been completed. If information was available on a specific indicator, data were extracted for further analysis. After the evaluation of each of the RE-AIM dimensions separately, the potential public health impact was assessed. Glasgow and colleagues state that the public health impact of an intervention depends on all five dimensions: reach, efficacy, adoption, implementation and maintenance. However in this review only three studies were included that reported on all five dimensions, so it was decided to lower the threshold from five to four dimensions: four studies reported on four dimensions. For each intervention, individual scores were calculated for reach (defined as the number of participants/number of eligible and invited people), efficacy (defined as the effect size of the intervention [30]), adoption (defined as the number of delivery settings/number of eligible and invited settings), implementation (defined as consistency of delivering intervention components) and maintenance (defined as the number of settings that maintained the intervention after the initial phase/number of settings that stopped delivering the intervention). Subsequently, the RE-AIM average was calculated by summing the scores on the five RE-AIM dimensions (or four if only four dimensions were available), and dividing them by four or five. These RE-AIM averages were considered to reflect the potential public health impact of the interventions [31,32].

## Results

### Study characteristics

**Figure 1:**
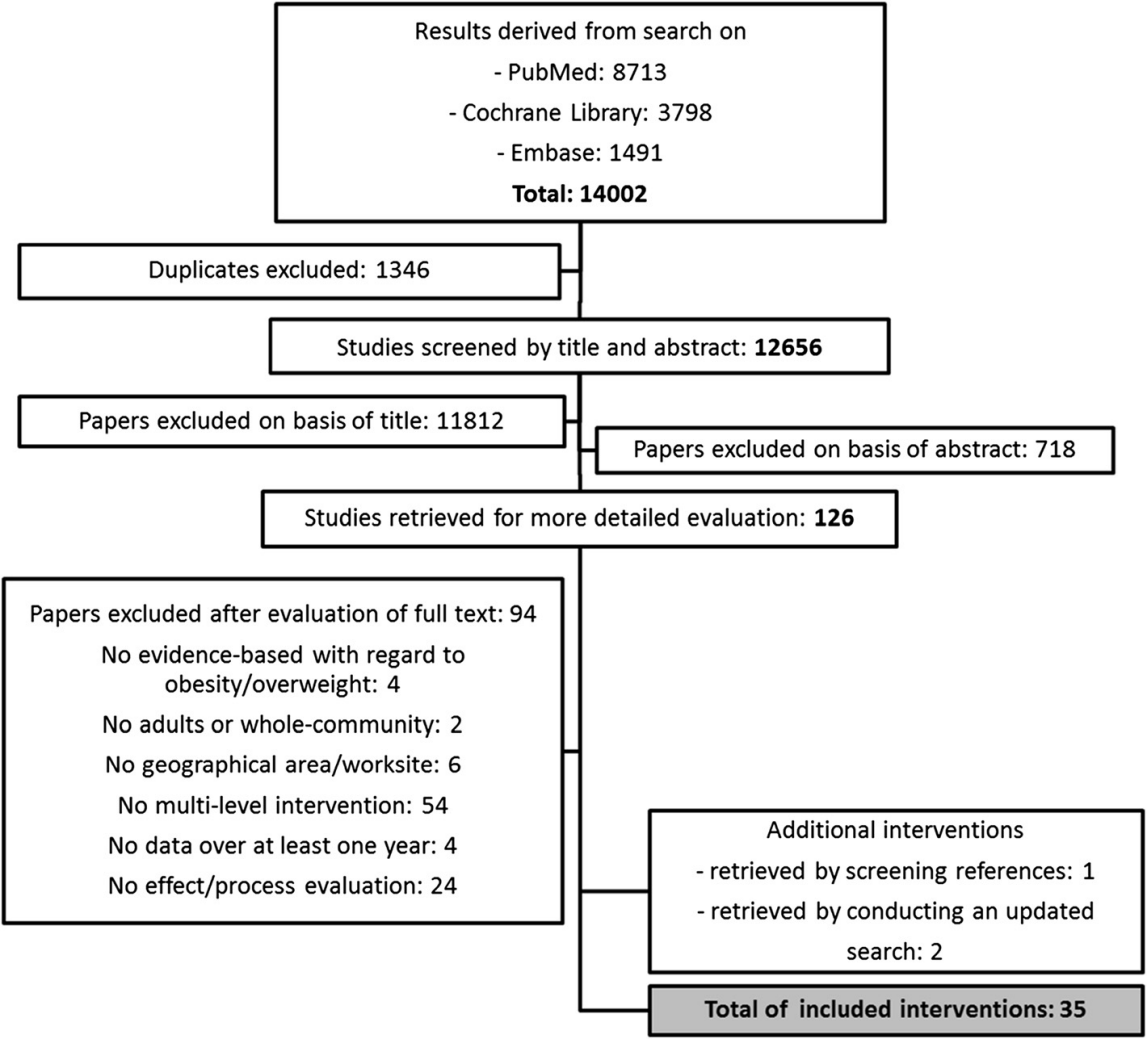
**Flow chart of study selection process.** Figure 1 provides an overview of the study selection process.

Of the 14,002 studies identified in the literature search in April 2012, 126 studies remained after removing duplicates and screening titles and abstracts. The full texts of the remaining studies were evaluated for the inclusion criteria, which resulted in a final selection of 33 interventions, described in 70 papers [33-102]. In October 2013, an update was conducted, which yielded another two interventions [103,104]. Consequently, 35 interventions were included in the systematic review. The flow chart in Figure 1 describes the entire selection process.

The characteristics of the identified studies are described in Additional file 2 and summarized in Table 1. The majority (69%) of the interventions were assessed by a cluster-randomized controlled trial [36-39,41,44,47,49,51-53,55,63,74,76,79-82,90,91,103]. Of the studies using a pre-experimental design (31%), 82% used a one group pretest posttest design without control group [35,50,60,67,75,87,93,100,102,104] and 18% used a one group only posttest design [68,92]. Different methods for data collection were used throughout the studies. In 32 studies [6,35-39,41,44,47,49-53,55,60,67,68,74,75,79-82,87,91-93,99,100,102-104], participants had to fill out a questionnaire to evaluate levels of physical activity and sedentary behaviour (n = 25), food intake (n = 19), knowledge on health and health-related behaviours (n = 8), psychosocial variables (n = 9) or awareness of/attendance at the intervention (n = 9). Two studies [44,51] utilized pedometers objectively to monitor levels of physical activity, and one study used direct observations to evaluate obesity-related behaviours. In 15 studies [35,36,47,51-53,55,61,64,74,79,84,87,91,104] clinical measurements were performed to determine overweight and obesity-related physiological outcomes, such as weight, height, blood pressure, waist circumference etc. The most commonly implemented individual-level intervention components were educational sessions (n = 11), individual counselling and advice on obesity-related behaviours (n = 9), posters (n = 8) and newsletters (n = 8). Other components were leaflets, websites, pedometers, logbooks, motivational messages, food and cooking demonstrations, education tours in supermarkets, maps with physical activity possibilities, individual feedback on clinical measurements and food labeling. The most commonly implemented environmental components were the establishment of walking and cycling groups (n = 14), the organization of physical activity group sessions (n = 13) and the increase of available healthy foods (n = 12). Other environmental intervention components were social support, the improvement of walking/cycling paths, the loan of pedometers, the increase of the number of physical activity areas and improved accessibility, the organization of health/physical activity events, the start of a physical activity competition, the reduction of prices of healthy food items, etc.

**Table 1:** **Characteristics of identified studies**

**Study characteristics**	**No. studies (%)**
**Design**	
Quasi-experimental design	70%
Pre-experimental design	30%
**Focus**	
Combination physical activity, sedentary behaviour and eating behaviour	57%
Physical activity	33%
Sedentary behaviour	0%
Eating behaviour	9%
**Setting**	
Schools/workplaces	27%
Churches	12%
Communities	61%
**Participants**	
> 1000 in *at least one* measurement	69%
> 1000 in *all* measurements	38%
**Data collection method**	
Questionnaire	91%
Clinical measures	39%
Pedometers	6%
**Geographical area**	
America	58%
Europe	18%
Oceania	15%
Asia	9%

### RE-AIM evaluation

Thirty-two interventions did not report on all five dimensions. More than one-third of the selected interventions (15/35) only provided information on the degree of effectiveness. Eight interventions confined themselves to the report of two dimensions, namely the reach and the effectiveness. Another eight interventions, reported on three dimensions, of which four reported on the effectiveness, the adoption and the implementation, three reported on the reach, the effectiveness and the adoption and one intervention reported on the reach, the effectiveness and the implementation. This left us with only four interventions that gave information on at least four of the five RE-AIM dimensions.

Table 2 presents the main results regarding the report on Reach, Effectiveness, Adoption, Implementation and Maintenance of the included interventions. Details of the information extracted from the intervention studies are provided in the paragraphs below (see Additional file 3).

**Table 2:** **Number of studies reporting on the different RE-AIM dimensions**

**Component**	**Number of studies reporting n (%)**
**Reach**	
- Description of target population within the geographical area/worksite	33 (100)
- Awareness of the intervention/participation rate	16 (48)
- Characteristics of people aware of the intervention/participants	10 (30)
- Representativeness of people aware of the intervention/participants	9 (27)
**Effectiveness**	
- Positive outcomes	30 (91)
- Quality of life	1 (3)
- Negative consequences	1 (3)
- Short-term attrition	15 (43)
**Adoption**	
- Description of staff delivering the intervention	6 (17)
- Representativeness of staff delivering the intervention	1 (3)
- Description of intervention delivery settings	23 (70)
- Description of non-delivering settings	5 (15)
- Representativeness of delivery settings	3 (9)
**Implementation**	
- Completeness of implementation	10 (30)
- Fidelity of implementation	2 (6)
- Time needed to implement the intervention	0 (0)
- Financial investment of the intervention	8 (24)
- Staff expertise of training of the deliverers	4 (12)
**Maintenance – setting**	
- Program sustainability	8 (24)
- Program adaptations	3 (9)
- Representativeness of organizations who are still delivering the intervention	1 (3)
**Maintenance – individual**	
- Long-term effects	8 (23)

### Reach

All studies described the target population of the intervention. Two interventions (6%) reported specific inclusion criteria, while all other interventions targeted the whole (adult) community/all employees. The number of people belonging to the target group was reported in 17 studies (49%) and varied from 500 to 37,000,000 people. Almost half of the studies (46%) gave information on the number of people affected by the intervention. The participation level of some intervention components could not be determined (e.g. improving street lightning, renovating walking paths, handing out flyers, putting up posters) so both awareness rates and participation rates were discussed. The mean awareness rate was 58%. Three studies [49,56,91] reported awareness rates above 90%, while one study [82] reported an awareness rate of less than 20%. In contrast to the high awareness rates, the mean participation rate in at least one activity was 30% and ranged from 1% in “Walk Kansas” [50] to 94% in “Body and Soul” [42]. The representativeness of people who were aware of the intervention/intervention participants was described in nine studies (26%). Of these nine studies, two found no significant differences, while seven observed significant differences by sex (n = 5; women were more likely to participate), age (n = 2; older people were more likely to participate), physical activity level (n = 2; active people were more likely to participate), BMI (n = 1; people with a higher BMI were more likely to participate) and ethnicity (n = 1; Western people were more likely to participate) between participants and non-participants.

### Effectiveness

Nearly all intervention studies (89%) recorded positive obesity-related behavioural (71%) or overweight and obesity-related physiological outcomes (34%). Of the studies reporting positive behavioural outcomes, seventeen reported on physical activity or sedentary behaviour, while thirteen studies reported on dietary behaviour. One study [102] notified a negative outcome and one study reported quality of life data [80]. Information on percent attrition was provided in fifteen studies, ranging from 4-85%.

### Adoption

Adoption of interventions was reported in all studies. At the staff level, six interventions described the intervention agents. Of these, five studies reported the number of intervention agents, ranging from 1–176, and five studies provided information on the characteristics of intervention agents. Only one study analysed the representativeness of intervention agents [97]. This analysis indicated that women and those with more years of experience of PA promotion are more likely to adopt the program than men and those with less experience of PA promotion. No significant differences were found in mean age between intervention agents and non-project staff members [97].

At the setting level, all the studies reported information on the delivery settings. Interventions were delivered in churches (n = 4), schools (n = 2), worksites (n = 8) and communities (n = 21). The number of delivery settings was reported in all worksite, school-based and church-based interventions, while only 50% of the community-based interventions gave information on the number of different delivery settings. The proportion of delivery settings was presented in 23% of the studies, and ranged from 9% (NHF-NRG In Balance [63]) to 92% (Walk Kansas [50]). Information on non-delivering settings was presented in 14% of the studies. Three studies compared the delivery setting with the non-delivery settings to work out their representativeness. This comparison showed significant differences between delivery settings and non-delivery settings in “Walk Kansas” and “Health-e-AME”. Both studies demonstrated that larger communities were more likely to adopt the intervention. No significant differences were found between deliverer settings and non-delivery settings in the intervention “10.000 Steps Flanders”.

### Implementation

Within implementation, the report of five items was evaluated. Completeness of implementation was reported in ten studies (29%). Three of these studies (10.000 Steps Flanders, Healthworks and Health-e-AME) gave a separate implementation score per intervention component. This implementation score reflected the percentage of intervention delivering settings that implemented a specific intervention component. In “10,000 Steps Flanders”, the implementation score varied from 17% for wide-ranging personal contact with citizens to 91% for the loan and sale of pedometers. “Healthworks” succeeded in implementing the offering of healthy food, the promotion of walking, the loan of pedometers, the dissemination of a newsletter and the promotion of stair use, but failed in achieving a reduction in healthy food prices. The implementation score of “Health-e-AME” components ranged from 7/50 for “8 Steps to Fitness” to 16/50 for the walking program. Reasons given for not implementing intervention components included ‘no time’, ‘too expensive’, no space’, ‘no added value for the project’, ‘not relevant to our core business’, and ‘lack of information to implement the component’. One study [64] ascribed the failure of implementation to external factors, such as vending drivers and food service managers, preventing food prices from being reduced due to concerns about possible adverse economic consequences. Fidelity of implementation was reported in two studies (6%), of which one [101] reported the adherence to program principles by component. None of the intervention studies reported time needed to implement the intervention; eight (23%) did not report financial investment of the organization, and four (11%) did not report staff expertise/training. Of the eight studies reporting on financial investment, seven emphasized the low costs for organizations, because they were sponsored by external grants, which varied from € 27,000 (Elementary School Personnel Intervention [91]) to € 900,000 (Hartslag Limburg [84]).

### Maintenance

Maintenance was subdivided by Glasgow et al. into the individual level maintenance and organizational level maintenance [26]. At the individual level, eight interventions reported their health behaviour at least six months beyond the study period. Of these, all studies found long-term effects. At the organizational level, to our knowledge, sixteen interventions were sustained until October 2013. Nevertheless, only eight studies explicitly described the continuation or dissemination of the intervention after the intervention study. Three studies evaluated the dissemination of the intervention and three studies reported on adaptations. The interventions “10,000 steps Flanders” [44] and “Agita São Paulo” [68] were not adapted after dissemination of the intervention, while “10,000 steps Rockhampton” [49] adapted the intervention through a website.

### Potential public health impact

**Figure 2:**
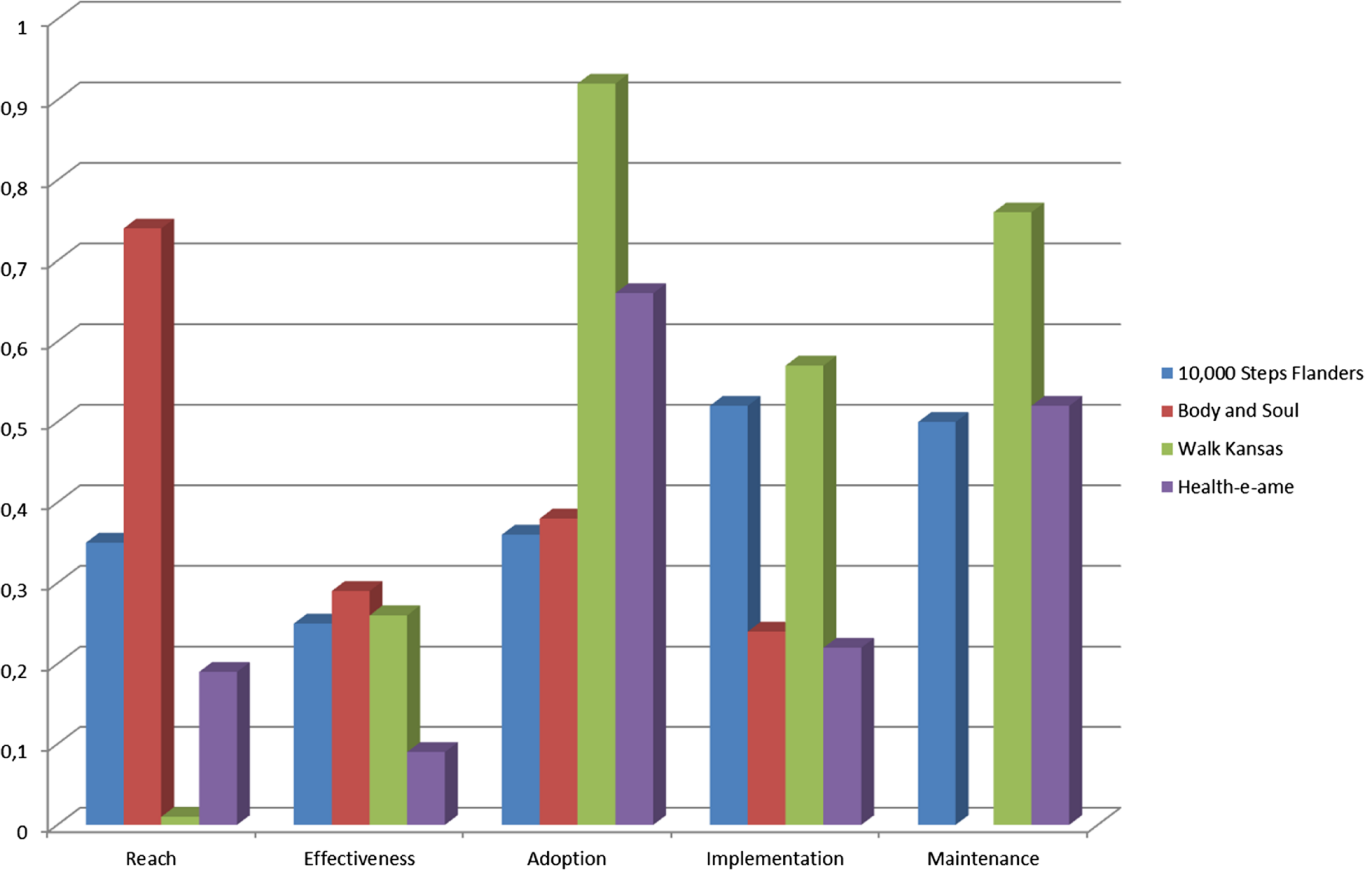
**Performance of 10,000 Flanders, Body and Soul, Walk Kansas and Health-e-ame on individual RE-AIM dimensions.** Figure 2 visually represents the performance of 10,000 Steps Flanders, Body and Soul, Walk Kansas and Health-e-AME on individual RE-AIM dimensions.

As mentioned above, the potential public health impact of four interventions was assessed (see Figure 2). Three of them provided information on all five dimensions: Walk Kansas (USA) [50], 10,000 Steps Flanders (Belgium) [44], and Health-e-AME (USA) [101], while one intervention provided information on four out of five dimensions: Body and Soul (USA) [81]. Both “Walk Kansas” and “10,000 Steps Flanders” were community-based physical activity programs, whereas the “Health-e-AME” intervention and the “Body and Soul” intervention were church-based interventions, focusing on physical activity and dietary behaviour, respectively. In “Walk Kansas”, participants formed a team and each team was supposed to identify a physical activity-related goal it wanted to reach. In “10,000 Steps Flanders”, several physical activity intervention components were implemented based on the central theme of reaching 10,000 steps/day. In “Health-e-AME, physical activity-related intervention components were implemented based on the Social Ecological Theory, and the Transtheoretical model, and in “Body and Soul”, intervention components related to healthy eating were implemented. When judging the potential public health impact of those four interventions by calculating the average RE-AIM score, the “Health-e-AME” intervention scored the lowest based on limited positive effects. Moreover, the different intervention components of the “Health-e-AME” intervention program were inconsistently implemented. This fragmented implementation was also observed for the “Body and Soul” intervention, in which only one out of eight intervention churches initiated all four pillars. In contrast, “10,000 Steps Flanders” noticed a modest global implementation score and “Walk Kansas” emphasized the consistent implementation of key intervention components. In spite of this, it cannot be presumed that “10,000 Steps Flanders” and “Walk Kansas” have a higher potential public health impact than “Health-e-AME” and “Body and Soul”, due to the restricted adoption rate of “10,000 Steps Flanders” (36%), and the limited participation rate of “Walk Kansas” (1%). Besides the adoption and participation rate, representativeness of participants and intervention agents was judged. In “10,000 Steps Flanders” no significant differences were found between participants and non-participants, whereas “Walk Kansas” and “Health-e-AME” identified that women were more likely to participate than men. In addition, “Walk Kansas” seemed more appealing for people who are already active, compared to non-active people. All four interventions found significant differences in representativeness of intervention deliverers: “10,000 Steps Flanders” concluded that staff members with longer experience of physical activity promotion were more likely to adopt the programme; “Health-e-AME” concluded that larger churches are more likely to adopt the programme; “Body and Soul” noticed that intervention deliverers were likely to have higher educational status and a higher income than non-deliverers. “Walk Kansas” was more often adopted by counties with higher populations. Sustainability was extensively discussed in three interventions: “10,000 Steps Flanders”, “Health-e-AME”, and “Walk Kansas”. “Walk Kansas” scored best, since 76% of the counties adopted the intervention for at least three years. Consequently, based on the RE-AIM evaluation, it can be concluded that “Walk Kansas” achieved the highest potential public health impact, in spite of its low participation rate.

## Discussion

The aim of this review was to provide an overview of the existing evidence-based multi-level interventions to improve obesity-related behaviours, and to determine their Reach, Effectiveness, Adoption, Implementation and Maintenance in order to assess their potential public health impact.

A total of 35 multi-level interventions were identified, described and assessed on the five RE-AIM dimensions. Typically, multi-level interventions are not evaluated using randomized controlled trials. This is due to the fact that multi-level interventions have components that cannot be individually randomized (e.g. environmental changes) and represent real-world settings. Despite their limitations conducting trials in real world settings provides information on whether an intervention works under usual conditions, which facilitates research translation [105].

Concerning the report of RE-AIM dimensions, the results showed that information was largely underreported, with description of elements of external validity and generalizability especially lacking. This finding is in line with the results of previous reviews [106-112] and hampers the assessment of potential public health impact, which is needed to determine if an intervention should be implemented and disseminated on a large scale.

Our findings on reach of the interventions are in accordance with the results reported in previous reviews [25,106-109]. As observed in the review of Vuillemin et al. [109], all studies described the intended target audience. This description was generally not very detailed, since the majority of the multi-level interventions were community-based, in which all members of the population were considered eligible. This is particularly relevant since people with health conditions are often omitted from experimental research [26,106,113]. Other aspects of reach were less frequently reported, although these aspects are important for assessing the external validity of the interventions. Only 46% of the studies provided information on the numbers of individuals actually reached, which is relatively low in comparison with the results of other reviews [106-108]. Overall participation rates were highest in church-based interventions, and lowest in community-based interventions. Furthermore, over half the studies declared that women were more likely to participate than men. Consequently, it seems that most of the obesity prevention approaches were less appealing to, or less adapted to the needs of, men rather than women.

In terms of Effectiveness of the interventions, positive overweight and obesity-related behavioural and, physiological outcomes are the most consistently reported aspects within the RE-AIM framework. However, these positive outcomes could be overestimated, since the majority of the studies did not account for attrition. Furthermore, negative outcomes of the intervention, and effects on quality of life are rarely reported, which is in agreement with the results of Dzewaltowksi et al. and Antikainen et al. [106,107]. Nonetheless, other reviews have identified higher percentages for reports of adverse consequences (30%-32%) [108,109]. The unbalanced relation in reporting positive and negative outcomes may be due to publication bias. However, knowledge on adverse effects is highly important for large-scale implementation and dissemination [26].

Regarding the Adoption of the interventions, similar to previous reviews [109,110], information on intervention agents was underreported. In contrast, the number of delivery settings was widely reported [106,108,109]. Unfortunately, this number provides insufficient information to assess the generalizability of an intervention. Therefore, our focus was on the adoption rate, which refers to the proportion of participating settings. The adoption rate was highest in “Walk Kansas”. However, “Walk Kansas” was developed based on strategies and principles about feasible implementation methods, whereas most other programs were developed simply and solely with attention to efficacy. Furthermore, most of the interventions describe the characteristics of the intervention deliverers, but only 14% of the interventions reported on characteristics of non-deliverers, which is comparable with the results of earlier reviews [106-109]. Consequently, information is lacking to assess the representativeness of the interventions, so no meaningful conclusions could be drawn with respect to the external validity of the interventions.

The first two aspects of implementation of the interventions - ‘completeness’ and ‘fidelity’ - are important to judge the internal validity of interventions and to assess the appropriateness of the interventions’ conclusions. In addition, reporting on the consistency of intervention components provides information on the degree of ease for implementing different components. Unfortunately, despite the relatively high number reporting on completeness or fidelity of implementation, the information was largely incomplete. Moreover, the ways of providing information on completeness varied considerably, so no comparison could be made between the interventions. Nonetheless, when looking at the reasons for not implementing intervention components, it can be concluded that intervention components need to be low-cost, time-efficient and suitable for organizations or communities with limited space. Moreover, the role of external factors needs to be reduced to a minimum so that the prosperity of an intervention component is independent of external factors. Furthermore, financial investment, time and expertise needed to implement the intervention were investigated in order to estimate the load for the intervention agents. Unlike previous reviews [108,109], only financial investment was extensively reported. However, it seems that the majority of the interventions were funded by external grants, so that no additional costs were required from the intervention deliverers.

Only eight intervention studies included information on maintenance in the form of programme sustainability, in spite of the continuation of sixteen interventions. However, this is a favourable result, compared to the results of previous reviews, in which programme sustainability varied between 0% and 5% [106,108,109]. Nevertheless, it should be acknowledged that all the included interventions were introduced in the last thirteen years, whereby the sustainability of the interventions is relative. Furthermore, it is notable that community –and church based interventions are more likely to be proceeded, than worksite –and school based interventions, which is in line with the results of Antikainen et al. [106]. They stated that all the studies that reported on institutional level maintenance were community-based interventions that focused on translating an intervention into a real-world setting.

Finally, the potential public health impact of four interventions that reported on at least four RE-AIM dimensions was evaluated. This evaluation was based on the RE-AIM average score defined by Glasgow et al. [31]. This score did not contain information on all aspects within the RE-AIM dimensions, whereby the score should be interpreted with caution. Of the four interventions [37,44,50,81], our findings suggest that “Walk Kansas” scored highest for potential public health impact. An important clarification for the high score of “Walk Kansas”, is that all levels were included, from potential program participants to organizational sponsors, at the beginning of the planning process. This resulted in an attractive program both for community members and programme deliverers. Moreover, in both “Walk Kansas” and “10,000 Steps Flanders”, the diffusion theory was used to guide the implementation process, which was defined as ‘the process by which an innovation is communicated through certain channels over time among the members of a social system’ [114]. Furthermore, both interventions used a website to inform potential participants, and to disseminate the intervention.

## Conclusions

The majority of the obesity-related multi-level intervention that we identified have the potential to reach a large amount of people, including those who can benefit most. Moreover, it seems that multi-level interventions are likely to be broadly adopted and to be sustained. RE-AIM assessment showed that multi-level interventions that 1) focused on all levels, from potential program participants to organizational sponsors, at the beginning of the planning process, 2) applied the diffusion theory to guide the implementation process, and 3) used a website to disseminate the intervention, achieved the highest potential public health impact. Nevertheless, better reporting of factors related to external validity and sustainability is needed to confirm these results.

## Additional files

Additional file 1:
**Search strategy.** Search strategy, existing of keywords used for inclusion, and keywords used for exclusion. Additional file 2:
**Characteristics of the included studies.** Characteristics of the identified studies, including information on the focus of the intervention, study design, target population, study participants, intervention components, outcome measures and geographical information. Additional file 3:
**RE-AIM evaluation.** Reach, Effectiveness, Adoption, Implementation and Maintenance of the included interventions.

## Abbreviations

RE-AIM: Reach, Effectiveness, Adoption, Implementation, Maintenance
