# Supplementary material for: A RE-AIM evaluation of evidence-based multi-level interventions to improve obesity-related behaviours in adults: a systematic review (the SPOTLIGHT project)
Source: Int J Behav Nutr Phys Act. 2014 Dec 6;11:147. doi: 10.1186/s12966-014-0147-3 (PMC4266878; doi:10.1186/s12966-014-0147-3)
Supplement: Additional file 1: — Search strategy. Search strategy, existing of keywords used for inclusion, and keywords used for exclusion. [file 12966_2014_147_MOESM1_ESM.pdf]

Additional file 1 – Search strategy

**Keywords used for inclusion**	
Population	adults OR employees OR men OR women OR community OR communities OR aged OR “older people” OR elderly OR “elder people” OR seniors
AND	
Intervention	program OR programme OR strategy OR strategies OR programs OR intervention OR promotion OR project
AND	
Outcome	“physical activity” OR exercise OR exercising OR sport OR sports OR walking OR cycling OR “active transport” OR commute OR “active commuting” OR “physical fitness” OR sedentary OR sitting OR nutrition OR food OR diet OR dietary OR BMI OR bodyweight OR overweight OR obesity OR “body composition” OR “body fat”
**Keywords used for exclusion**	
NOT	
breastfeeding OR pregnant OR pregnancy OR alcohol OR treat OR treatment OR therapy OR therapeutic OR serum OR “back pain” OR treating OR cancer OR carcinoma OR osteoporosis OR osteoporotic OR bone OR fibromyalgia OR “vitamin D” OR anemia	
