# Supplementary material for: A RE-AIM evaluation of evidence-based multi-level interventions to improve obesity-related behaviours in adults: a systematic review (the SPOTLIGHT project)
Source: Int J Behav Nutr Phys Act. 2014 Dec 6;11:147. doi: 10.1186/s12966-014-0147-3 (PMC4266878; doi:10.1186/s12966-014-0147-3)
Supplement: Additional file 2: — Characteristics of the included studies. Characteristics of the identified studies, including information on the focus of the intervention, study design, target population, study participants, intervention components, outcome measures and geographical information. [file 12966_2014_147_MOESM2_ESM.pdf]

Additional file 2: Characteristics of the included studies

Reference (Name intervention, Author, Date)	Focus (PA, SB, EB*)	Study design	Target population	Study participants (I, C**) (n)	Environmental intervention components	Individual intervention components	Outcome measurements	European (E) or Non-European Study (NE)***
**1.Romsas in Motion**<br>Jenum A.K. et al.; 2003<br>Jenum A.K. et al.; 2006<br>Lorentzen C. et al.; 2007<br>Jenum A.K. et al.; 2009	PA	Cluster-randomised controlled design	Age: 30 – 67 years<br>I: Romsas<br>C: Furuset	Baseline (2000):<br>I: 1497 participants<br>C: 1453 participants<br>Follow-up (2003):<br>I: 890 participants<br>C: 876 participants	- Walking groups<br>- Group sessions for indoor activity at no cost<br>- Social support by the district administration staff<br>- Labeling of walking trails<br>- Improving street lighting<br>- Gritting of pavements and trials in the winter	- Leaflets<br>- Reminders of the health benefits of using the stairs<br>- Local meetings about health<br>- Individual counseling	- Health status, self-reported disease, health-related behaviour (questionnaire)<br>- Body height, weight, waist and hip circumferences, blood pressure, cholesterol, glucose, lipids (clinical measurements)	E: Norway, Oslo
**2. 10.000 steps Flanders**<br>De Cocker K.A. et al.; 2007<br>Van Acker R. et al.; 2011<br>De Cocker K.A. et al.; 2011<br>Van Acker R. et al.; 2012<br>Dubuy V. et al. ; 2013	PA	Cluster-randomised controlled design	Age: 25 – 75 years<br>I: Ghent<br>C: Aalst	Baseline (2005):<br>I: 872 participants<br>C: 810 participants<br>Follow-up1 (2006):<br>I: 660 participants<br>C: 634 participants<br>Follow-up2 (2009):<br>I: 216 participants<br>C: 204 participants	- Street signs, walking routes<br>- Sale and loan of pedometers	- Website<br>- Local media project<br>- Flyers, posters etc.	- PA (questionnaire: IPAQ)<br>- Steps (pedometer + activity log)<br>- Awareness of the 10.000 steps Ghent (questionnaire)	E: Belgium; Ghent, Flanders
**3. 10.000 steps Rockhampton**<br>Brown W.J. et ak.; 2006<br>Eakin E.G. et al. ; 2007<br>Mummery W.K. et al.; 2006	PA	Cluster-randomised controlled design	Age > 18 years<br>I: Rockhampton<br>C: Mackay	Baseline (2001):<br>I: 1280 participants<br>C: 1059 participants<br>Follow-up (2003):<br>I: 1242 participants<br>C: 1236 participants	- Improve the local environment, e.g. repairing key footpaths, erecting 10,000 steps signs	- Loan of pedometers<br>- Logbooks are available<br>- Print, radio and TV media campaign<br>- Advice of general practitioners	- Changes in physical activity (questionnaire: Active Australia Survey)<br>- Awareness of the 10,000 steps project (questionnaire)<br>- Pedometer use (questionnaire)	NE: Australia, Rockhamton
**4. Isfahan Healthy Heart Programme**	PA and EB	Cluster-randomised	Age range not specified	Baseline (2000):<br>I: 6175 participants	- Development of public exercise areas and green	- Campaigns and multi-media, education of	- Physical activity (questionnaire)	NE: Iran, Isfahan

Sarrafzadegan N. et al. ; 2003<br>Sarrafzadegan N. et al. ; 2008<br>Rabiei K. et al. ; 2009<br>Rabiei K. et al. ; 2010<br>Boshtam M. et al. ; 2010<br>Khosravi A. et al. ; 2010<br>Baghaei A. et al. ; 2010<br>Tavasolli A. et al. ; 2011		controlled design8	I: Isfahan and Najafabad<br>C: Arak	C: 6339 participants<br>Follow-up1 (2001):<br>I: 2994 participants<br>C: 2897 participants<br>Follow-up2 (2002):<br>I: 2400 participants<br>C: 2393 participants<br>Follow-up3 (2003):<br>I: 3012 participants<br>C: 3070 participants<br>Follow-up4 (2004):<br>I: 3011 participants<br>C: NA	spaces<br>- Increase in the number of sport centers<br>- Development of the infrastructures required for reducing the use of personal motor vehicles and encouraging cycling and walking<br>- Modification and improvement of urban environments	specific target groups, face-to-face education, class education	- Dietary intake (questionnaire : FFQ)<br>- Smoking behaviour (questionnaire)	
**5. The Agita São Paulo Program**<br>Matsudo V. et al. ; 2002<br>Matsudo S.M. et al. ; 2003<br>Matsudo S.M. et al. ; 2005	PA	Pre-experimental design: one group posttest only design	Age range not specified<br>I: São Paulo	I : Metropolitan (1999) : 641 participants<br>(2000) : 645 participants<br>(2001) : 627 participants<br>(2002) : 662 participants<br>(2003): 651 participants<br><br>I: SP State (2002): 2001 participants<br>(2003): 2000 participants	- Mega-events: Agita Trabalhador, Agita Galera, Agita Melhoridade<br>- Changes to physical spaces: greater access to sporting facilities , creating more green and leisure spaces	- Posters, flyers<br>- Messages in newsletters, magazines of organizations<br>- Mascot<br>- Media messages<br>- Agital: package containing educational materials on physical activity for health professionals to prescribe to patients	- Physical activity level (questionnaire: IPAQ)<br>- Physical activity knowledge (questionnaire)<br>- Behaviour stage (questionnaire)<br>- Recall of the program (questionnaire)	NE: Brazil, São Paulo
**6. The Healthy Hawaii Initiative**<br>Nigg C. et al.; 2005<br>Maddock J. et al.; 2006	PA and EB	Pre-experimental design: one group pretest posttest design	Age range not specified<br>I: Hawaii	Baseline (1999) :<br>I : NA<br>Follow-up1 (2000) :<br>I : NA<br>Follow-up2 (2001) :<br>I : NA<br>Follow-up3 (2002) :<br>I : 4648 participants<br>Follow-up4 (2003) :	- Renovating walking paths<br>- Changing health screenings<br>- Making physical venues for older adults<br>- Increasing healthy choices in restaurants	- Public education campaign 'Start Living Healthy' with press releases, radio advertisements, a website	- Mortality rate, diabetes prevalence, obese and overweight prevalence (questionnaire)<br>- Fruit and vegetables intake, physical activity (questionnaire: BRFSS and YRBS)<br>- Perceived environment,	NE: Hawaii

				I : 4495 participants<br>Follow-up5 (2004) :<br>I : 4476 participants			attitudes, subjective norms, stage-of-change, self-efficacy (questionnaire)	
**7. Walk the Ozarks to Welness**<br>Brownson R.C. et al.; 2004<br>Brownson R.C. et al. ; 2005	PA	Cluster-randomised controlled design	Age > 18 years<br>I: Six rural communities in Missouri<br>C: Six rural communities in Arkansas en Tennessee	Baseline (2003):<br>I: NA<br>C: NA<br>Follow-up (2004):<br>I: 752 participants<br>C: 779 participants	- Walking clubs<br>- Activities such as walk-a-thons	- Computer-based tailoring<br>- Tailored newsletters<br>- Feedback letters by mail<br>- Counseling	- Physical activity, being aware of project, attending a project WOW event, sometimes or often seeing signs, articles, or other media related to PA, reporting that his physician advised him to exercise more (questionnaire: BRFSS)	NE: United States, Missouri, Arkansas and Tennessee
**8. Healthworks**<br>Linde A.J. et al.; 2012	PA and EB	Cluster-randomised controlled design	Age: 18-75 years<br>I: three worksite in the Twin Cities metropolitan area<br>C: three worksite in the Twin Cities metropolitan area	Baseline (2006 2007):<br>I: 752 participants<br>C: 995 participants<br>Follow-up (2008-2009):<br>I: 611 participants<br>C: 795 participants	- Organized group walks<br>- Competition between co-workers<br>- Weight tracking competitions<br>- Increase the availability of calorie smart foods<br>- Reduce the price of calorie smart foods<br>- Offer small portion sizes as substitutes	- Label calorie smart items<br>- Activity monitoring (pedometer)<br>- Motivation signs, posters, music were places in select stairwells<br>- Balance beam scales with BMI charts<br>- Monthly newsletter	- Weight, BMI (clinical measurements)<br>- Food inventory (observational study)<br>- Stair use (infrared beam sensors)<br>- Health media environment (observational study)	NE: US; Minnesota
**9. Hartslag Limburg**<br>Ronda G. et al. ; 2004<br>Ronda G. et al. ; 2004<br>Ronda G. et al. ; 2005<br>Schuit A.J. et al. ; 2006<br>Ronckers E.T. et al. ; 2006<br>Verkleij S.P. et al. ; 2011	PA and EB	Cluster-randomised controlled design	Age : 20 – 59 years<br>I : Maastricht<br>C : NA	Baseline (1998) :<br>I : 3000 participants<br>C : 895 participants<br>Follow-up (2003) :<br>I : 2414 participants<br>C : 758 participants	- Public-private collaboration with the retail sector<br>- Walking and bicycling clubs	- Nutrition education classes<br>- Nutrition education tours in supermarkets<br>- Food labeling	- Blood pressure, height, weight, BMI, waist circumference, total cholesterol, HDL cholesterol, glucose concentration (Clinical measurement)	E : The Netherlands, Maastricht

**10. The Well London Project**<br>Wall M. et al. ; 2009<br>Philips G. et al. ; 2012	PA and EB	Cluster-randomised controlled design	Age >11 years (adults and adolescents)<br>I : 20 deprived neighborhoods in London<br>C : 20 deprived neighborhoods in London	Baseline (2008) :<br>I : 2061 adults and 618 adolescents<br>C : 2046 adults and 596 adolescents<br>Follow-up (2012) : NA	- Improve the quality of public spaces to encourage physical activity<br>- Improve access to healthy food choices	- Maps informing participants of local sources for making healthy choices<br>- Project to raise awareness of the importance of diet etc.	- Healthy eating, physical activity, mental wellbeing (questionnaires ; FFQ, IPAQ, PAQ-A Hope Scale, EQ5D, Strengths and Difficulties questionnaire and Positive and Negative Affect Scale)	E : United Kingdom, London
**11. Looma Healthy Lifestyle Program**<br>Rowley K.G. et al. ; 2000	PA and EB	Pre-experimental design: one group pretest posttest design	Age > 15 years<br>I : Looma Aboriginal community	Baseline (1993-1994) :<br>I : 199 participants<br>Follow-up1 (1996) :<br>I : 181 participants<br>Follow-up2 (1997) :<br>I : 125 participants	- Improved quality and quantity of fresh products<br>- Replacement of butter with margarine<br>- Avalaibility of wholemeal bread and flour<br>- Family walking groups<br>- Activation of sporting teams<br>- Sporting festivals	- Nutrition education<br>- Healthy cooking classes<br>- Store tours to identify healthy food choices	- BMI, fasting glucose, insulin, triglyceride (clinical measurements)<br>- Physical activity and dietary habits (questionnaire)	NE : Australia, Kimberley
**12. Healthy Employee Lifestyle Program**<br>Perez A.P. et al. ; 2009	EB	Pre-experimental design: one group pretest posttest design	Age range not specified<br>I : Employees of the Arkansas Department of Health	Baseline (2005) :<br>I : 1017 participants<br>Follow-up (2006) :<br>I : 214 participants	- Possibility to redeem the earned points for rewards (e.g. water bottles, 3 days of paid leave)	- Educational information (flyers, posters)<br>- Health promotion activities<br>- Health events	- Intake frequency of fat, desserts, protein, grains, dairy, processed meat, fried foods (questionnaire)<br>- Stage of change (questionnaire)	NE: USA, Arkansas
**13. Cycling connecting communities**<br>Rissel C.E. et al. ; 2010	PA	Cluster-randomised controlled design	Age > 18 years<br>I: Fairfield and Liverpool<br>C: Bankstown	Baseline (2007):<br>I + C: 1450 participants<br>Follow-up (2009):<br>I: 520 participants<br>C: 389 participants	- Community rides<br>- Free cycle skill courses	- ‘Discover Fairfield and Liverpool by bike’ map for illustrating the extent of local bike paths<br>- ‘Thinking about cycling’<br>- Presentation about the project	- Frequency of cycling, PA behaviour, usage of bicycle paths (questionnaire)<br>- Use of bicycle paths (observational study)	NE: Australia, Sydney
**14. Concord, a great place to be active**<br>Wen L.M. et al.; 2002	PA	Pre-experimental design: one group pretest posttest design	Age: 20-50 years<br>Women<br>I: Concord Local Government	Baseline (1997):<br>I: 1762 participants<br>Follow-up (1999):<br>I: 1801 participants	- Walking groups<br>- Community walking events<br>- Signage<br>- Community physical activity classes	- Media<br>- Newsletters<br>- Walking maps	- PA level (questionnaire)	NE: Australia, Sydney

			Area					
**15. The NHF-NRG In Balance Project**<br>Kwak L. et al.; 2006<br>Kwak L. et al.; 2009<br>Kwak L. et al.; 2010	PA and SB	Cluster-randomised controlled design	Age < 40 year<br>BMI > 18 kg/m²<br>I: 6 Dutch worksites<br>C: 6 Dutch worksites	Baseline (2003)<br>I: 365 participants<br>C: 188 participants<br>Follow-up1 (2004)<br>I: 294 participants<br>C: 164 participants<br>Follow-up2 (2005-2006)<br>I: 255 participants<br>C: 145 participants	- Form lunch walking groups<br>- Form employee advisory board<br>- Form groups of colleagues that actively commute together<br>- Increase distance from individual offices to printers, faxes, coffee machines etc.<br>- Facilitate of improve shower/change facilities<br>- Facilitate or improve bike sheds<br>- Bike-to-work campaign	- Feedback on measured BMI, fat percentage and waist circumference<br>- In-balance-box: pedometer, waist circumference measuring tape, calorie guide and instruction brochure<br>- In-Balance website<br>- Computer-tailored CD-rom<br>- Posters and leaflets	- Body weight, height, skinfold thickness and waist circumference (clinical measurements)	E: The Netherlands
**16. Familias Sanas y Activas**<br>Ayala G.X. et al. ; 2011	PA	Pre-experimental design: one group pretest posttest design	Age > 18 years<br>Women<br>I: South San Diego county	Baseline (2007):<br>I: 337 participants<br>Follow-up1 (2008):<br>I: participants<br>Follow-up2 (2008-2009):<br>I: 207 participants	- Incentives for participants: a pedometer, water bottle, stretch band etc.<br>- Free exercise classes in public places	- PA promotional material (e.g. community resource guide of free and low-cost PA resources)<br>- Walking path maps	- Sitting resting blood pressure, waist circumference, weight, height (clinical measurements)<br>- Leg and hamstrings flexibility (Sit-and-reach test)<br>- Aerobic fitness (6-minute walk test)<br>- Physical activity (questionnaire ; GPAQ)<br>- Psychosocial variables (questionnaire ; GRAD)	NE: USA, San Diego County
**17. Samoan communities church intervention**<br>Bell A.C. at al.; 2001	PA and EB	Cluster-randomised controlled design	Age: 20-77 years<br>Samoan church community members<br>I: Otara<br>C: Glen Innes and Glen Eden	Baseline (1995-1996):<br>I: 365 participants<br>C: 106 participants<br>Follow-up (1996):<br>I: 275 participants<br>C: 71 participants	- Aerobic sessions<br>- Walking clubs	- Nutrition education sessions	- Diet and lifestyle behaviour (questionnaire)<br>- Fat knowledge (questionnaire)<br>- PA (questionnaire)<br>- Anthropometric measurement and blood pressure (clinical measurements)	NE: New-Zealand, Otara, Glen Innes and Glen Eden
**18. Health-e-AME**<br>Bopp M. et al.; 2007	PA	Cluster-randomised	Age > 18 years<br>AME church	Baseline (2002):<br>I: 311 participants	- Praise aerobics<br>- Walking programs	- Educational messages	- Physical activity behaviour (questionnaire;	NE: South Carolina

Wilcox S. et al.; 2007<br>Bopp M. et al.; 2009		controlled design	members<br>I: eleven AME churches<br>C: nine AME churches	C: 260 participants<br>Follow-up1:<br>418 participants<br>Follow-up2:<br>316 participants	- Health directors or physical activity coordinators		BRFSS)<br>- Health-related variables (questionnaire)	
**19. Body and Soul**<br>Resnicow K. et al.; 2004<br>Campbell M.K. et al. ; 2007<br>Allicock M. et al.; 2012	EB	Cluster-randomised controlled design	Age > 18 years<br>African American church members	Baseline:<br>I+ C: 1022 participants<br>Follow-up:<br>I+C: 864 participants	- Pastor support<br>- Motivational interviewing	- Nutrition events<br>- Food demonstrations<br>- Supermarket tours<br>- Information sessions<br>- Self-help materials (e.g. cookbook)	- Fruit and vegetable intake (questionnaire; FFQ)<br>- Fat intake (questionnaire; NCI Fat Screener)<br>- Vegetable preparation practices (questionnaire)<br>- Intrinsic, extrinsic motivation, social support, self-efficacy (questionnaire)<br>- ¨Program exposure and attendance (questionnaire)	NE: USA, North Carolina
**20. Walk Kansas**<br>Estabrooks P.A. et al.; 2008	PA	Pre-experimental design: one group pretest posttest design	Age > 18 years<br>I: Kansas	Baseline:<br>I: 1045 participants<br>Follow-up:<br>I: 900 participants<br>Follow-up 6 month after completion of the program:<br>I: 278 participants	- Social support (team competition)	- Weekly newsletter<br>- Goal setting<br>- Healthy tips<br>- Celebration event	- PA (questionnaire, BRFSS)	NE: USA, Kansas
**21. Worksite intervention among Metropolitan Transit Workers**<br>French S.A. et al. ; 2010	PA and EB	Cluster-randomised controlled design	Age range not specified<br>Metropolitan Transit Workers	Baseline (2005):<br>I+C: 1102 participants<br>Follow-up (2007):<br>I+C: 940 participants	- Increased availability of healthful food and beverage in vending machines<br>- Lower prices for healthy food<br>- Fitness facilities<br>- Self-weighting team competition<br>- Team walking competition	- A free one-day health expo was held to increase awareness	- Weight and height (clinical measurement)<br>- Dietary intake, physical activity (questionnaire, FFQ, dietary recall, Godin leisure time PA and accelerometry)<br>- Perceived worksite environment and social support (questionnaire)	NE: USA, Mineapolis
**22. Merrill Lynch Intervention**<br>Gemson D.H. et al.; 2008	PA and EB	Cluster-randomised controlled design	Age range not specified<br>Employees with high blood pressure of Merrill Lynch	Baseline (2004):<br>I: 298 participants<br>C: 255 participants<br>Follow-up (2005):<br>I: 47 participants<br>C: 94 participants	- Variety of fruits in the cafeteria	- Screening program<br>- Health information<br>- Educational brochures<br>- Pedometers<br>- Information of nurses	- Blood pressure (clinical measurements)<br>- PA, diet and nutrition behaviours, pedometer use, weight, and motivation to change (questionnaire)	NE: USA

			Company					
**23. Dow Chemical Company Intervention**<br>Goetzel R.Z. et al.; 2009<br>Goetzel R.Z. et al.; 2010	PA and EB	Cluster-randomised controlled design	Age range not specified<br>Employees of Dow Chemical Company	Baseline (2006):<br>I: 4231 participants (health risk assessment)<br>2924 participants (biometric measurements)<br>C: 978 participants (health risk assessment)<br>646 participants (biometric measurements)<br>Follow-up (2007):<br>I: 2518 participants (health risk assessment)<br>1583 participants (biometric measurements)<br>C: 634 participants (health risk assessment)<br>417 participants (biometric measurements)	- Environmental prompts to encourage healthy food choices and PA behaviour<br>- Modifying vending machine items and cafeteria menus<br>- Creating walking paths	- Health education material (newsletters, posters, mailing, etc.)<br>- PA and weight management counseling<br>- Health assessment	- Health behaviours (questionnaire)<br>- Biometric screening measurement: height, weight, blood pressure (clinical measurements)	NE: USA, Texas, Louisiana, New Jersey, West Virginia
**24. Eating for a Healthy Life**<br>Hannon P.A. et al.; 2008<br>Bowen D.J. et al.; 2009	EB	Cluster-randomised controlled design	Age > 18 years<br>Members of faith communities	Baseline:<br>I: 1099 participants<br>C: 1076 participants<br>Follow-up:<br>I: 996 participants<br>C: 959 participants	- Social activities	- Healthful eating sessions (educational classes)<br>- Motivational messages	- Dietary behaviour (questionnaire; Fat and Fiber Behaviour Questionnaire and 24-hour dietary recall)<br>- Stages of change, perceived health (questionnaire)	NE: Seattle, Washington
**25. Worksite Opportunities for Welness (WOW)**<br>Racette S.B. et al.; 2009	PA and EB	Cluster-randomised controlled design	Age > 18 years<br>Employees of a large medical center in St.-Louis	Baseline (2005):<br>C: 67 participants<br>I: 84 participants<br>Follow-up (2006):<br>C: 55 participants<br>I: 68 participants	- Weekly healthy snack card<br>- Exercise program<br>- Team competition<br>- Participation rewards	- Pedometers<br>- One site Weight-Watchers group meetings<br>- Newsletters<br>- Walking maps	- Health assessment: height, weight, waist circumference, body composition, resting heart rate, blood pressure, fasting lipids and glucose, cardiovascular fitness (clinical measurements)	NE: USA, Missouri

							- Dietary and PA patterns (questionnaires; National Institutes of health fruit and vegetable screener, The Kristal Fat and Fiber Behaviour Questionnaire and the IPAQ)	
**26. Community intervention Bangladesh** Rana A.K.M. et al.; 2009	PA and EB	Cluster-randomised controlled design	Age > 60 years<br>I: 4 rural communities in Bangladesh<br>C: 4 rural communities in Bangladesh	Baseline (2003):<br>C: 517 participants<br>I: 514 participants<br>Follow-up (2004):<br>C: 414 participants<br>I: 425 participants	- Training on physical activity<br>- Social support	- Advice to avoid harmful food items<br>- Posters and leaflets to raise awareness	- Health related quality of life (questionnaire)	NE: Bangladesh
**27. Elementary school personnel intervention** Siegel J.M. et al.; 2010	PA and EB	Cluster-randomised controlled design	Age range not specified<br>I: Personnel of 8 schools<br>C: Personnel of 8 schools	Baseline (2005):<br>C: 288 participants<br>I: 145 participants<br>Follow-up (2007):<br>C: 125 participants<br>I: 66 participants	- Healthy snacks at meetings<br>- Walking clubs<br>- Interschool competition (with prizes)	- Newsletter<br>- Healthy cooking classes	- Weight, height, waist – and hip circumferences (clinical measurements)<br>- Physical activity and fruit and vegetables consumption (questionnaires: IPAQ and NCI all day screener)	NE: Los Angeles, California
**28. Academia Da Cidade** Simoes E.J. et al.; 2009	PA and EB	Pre-experimental design: one group posttest only design	Age > 16 years<br>Residents of Recife	(2007):<br>2046 participants	- Free supervised PA lessons	- Nutrition education<br>- Health monitoring	- Leisure time PA and transport PA (questionnaire, IPAQ)<br>- Participation and awareness of the activities (questionnaire)	NE: Recife, Brazilia
**29. Beijing University Inytervention** Xiangyang T. et al.; 2003	PA and EB	Pre-experimental design: one group pretest posttest design	Age range not specified<br>Employees and students of Beijing University	Baseline (1999):<br>2360 participants<br>Follow-up (2000):<br>2347 participants	- Workshops to develop healthy policies (e.g. provision of healthy food, routine annual physical examination etc.)	- Booklets, lecturers, interactive arena for health consultations, etc.	- Health knowledge, lifestyle behaviours: physical activity, smoking, dietary behaviour, sexual intercourse and mental health (questionnaire)	NE: Beijing
**30. Heart at Work** Pegus C. et al.; 2002	PA and EB	Cluster-randomised controlled design	Age range not specified<br>Employees of American worksites	Baseline (1998):<br>I: NA<br>C: NA<br>follow-up (1999):<br>I: 223 participants<br>C: 410 participants	- Walking group led by an coordinator who encourages the participants<br>- Health care coordinator<br>- Walking track was constructed on the	- E-mails messages and memos, flyers and posters,<br>- Small incentives: T-shirts, bags etc.<br>- Information about how to reduce fat intake	- Knowledge of CVD risk factors, nutrition and diet, self-efficacy (questionnaire)<br>- PA, weight management, CVD-related diet and nutrition and tobacco use (questionnaire)<br>- Cholesterol, blood	NE: USA

					factory grounds<br>- Vending machines stocked more low-fat snacks<br>- Low-fat lunch option		pressure (clinical measurements)	
**31. Pasos Adelante**<br>Staten L.K. et al.; 2005	PA and EB	Pre-experimental design: one group pretest posttest design	Age range not specified<br>I: Yuma and Santa Cruz counties	Baseline (2000-2003):<br>248 participants<br>Follow-up (2000-2003):<br>216 participants	- Walking club/group	- Lectures on health<br>- Educational classroom session on health	- Food intake and physical activity (questionnaires; Behavioural Risk Factor Surveillance Survey and Minnesota Leisure Time Physical Activity Questionnaire)	NE: USA; Arizona, United States-Sonora, Mexico border counties
**32. Wijkgezondheids-werk – Gezond in de buurt**<br>Kloek G.C. et al.; 2006<br>Kloek G.C. et al.; 2006	PA and EB	Cluster-randomised controlled design	Age: 18-65 years<br>I: Tivoli and De Bennekel (deprived neighborhoods in Eindhoven)<br>C: Three comparison neighborhoods in Eindhoven	Baseline (2000):<br>I: 1426<br>C: 1355<br>Follow-up (2002):<br>I: 1021<br>C: 908	- Neighborhoods walking tours<br>- Gymnastic classes<br>- Large community events related to health<br>- Collaboration with the greengrocer's shop	- Information on healthy nutrition	- Intervention awareness (questionnaire)<br>- Daily fruit and vegetable intake and physical activity (questionnaires: Food Frequency Questionnaire, SQUASH)<br>- Attitude, self-efficacy expectations, awareness, stages of change (questionnaire)	E: The Netherlands; Eindhoven
**33. The Groninger Active Living Model**<br>Stevens M. et al.; 2008<br>De Jong J. et al.; 2006<br>De Jong J. et al.; 2007<br>Stevens M. et al.; 2003	PA	Cluster-randomised controlled trial	Age: 55-65 years<br>I: 6 neighborhoord s in 3 Dutch municipalities<br>C: 6 neighborhoord s in 3 Dutch municipalities	Baseline (2000):<br>I: 163<br>C: 152<br>Follow-up (2001):<br>I: 79<br>C: 102	- Group physical activity sessions	- Sport and exercise advice	- Physical activity (Voorrips Physical Activity questionnaire)<br>- Perceived fitness (Groningen Fitness test for elderly questionnaire)<br>- Fitness (Groninger Fitness test)<br>- Blood pressure, body fat and BMI (clinical measures)	E: The Netherlands
**34. The Navajo Healthy Stores Program**<br>Gittelsohn J. et al.; 2013	EB	Cluster-randomised controlled trial	Age: ≥ 18 years<br>I+C: 10 store regions in the Navaja Nation	Baseline:<br>I+C: 276<br>Follow-up:<br>I+C: 145	- Increase availability of healthy food	- Promotion of healthy foods<br>- Demonstrating healthier cooking methods	- Attitudes, knowledge and dietary behaviour (Adult Impact Questionnaire)<br>- Intervention exposure (Intervention Exposure Questionnaire)	NE: USA, Arizona

**35. The Healthy Alberta Communities** Raine K.D.; 2013	PA and EB	Pre-experimental design: one group pretest posttest design	Age: ≥ 18 years<br>I: 4 communities in Alberta<br>C: all other communities in Alberta	Baseline (2006):<br>I: 4761<br>C: 9775<br>Follow-up (2009):<br>I: 4733<br>C: 9784	- Increase access to fruit and vegetables<br>- Increase accessibility of community recreation space<br>-Increase healthy restaurant options<br>- Increase opportunities for unstructured physical activity<br>-Increase the capacity of social programs	- Promotion of physical activity and healthy food items	- Health status, physical activity and dietary behaviour, height and weight (Canadian Community Health Survey)<br>-Anthropometric measures and blood pressure (clinical measures)<br>- Cholesterol, glucose, lipoprotein and triglycerides (blood sample)	NE: Canada, Alberta

* PA = physical activity; SB = sedentary behaviour; EB = eating behaviour
** I = Intervention group; C = control group
*** E = European study; NE = Non-European study
