# Supplementary material for: A RE-AIM evaluation of evidence-based multi-level interventions to improve obesity-related behaviours in adults: a systematic review (the SPOTLIGHT project)
Source: Int J Behav Nutr Phys Act. 2014 Dec 6;11:147. doi: 10.1186/s12966-014-0147-3 (PMC4266878; doi:10.1186/s12966-014-0147-3)
Supplement: Additional file 3: — RE-AIM evaluation. Reach, Effectiveness, Adoption, Implementation and Maintenance of the included interventions. [file 12966_2014_147_MOESM3_ESM.pdf]

	Reach		Efficacy	Adoption		Implementation	Maintenance	
	Awareness rate (A) / Participation rate (PR)	Representativeness: Difference (D) No difference (ND)	Psychosocial effects (PSE) Behavioral effects (BE) Physiological effects (PE)	Adoption rate (AR) and representativeness of intervention agents	Adoption rate of delivery setting(AR) and representativeness of delivery settings: Difference (D) No difference(ND)	Completeness / fidelity of implementation	Individual level maintenance	Program sustainability
**1.Romsas in Motion**	**Awareness**: 95,3%<br>**Participation**: 3,7% (lectures), 9,9% (walking groups), 13,3% (aerobic exercise groups), 21,7% (walk path), 6,4% (walk diary), 50,8% (physical fitness test)	**Awareness:**<br>**D**: Sign. more active, Western people, with high BMI noticed the project<br>**ND**: Gender, age and education<br>**Participation:**<br>**D**: Highly educated people are more likely to participate in the physical fitness test<br>**D**: Older people are more likely to participate in walking groups<br>**D**: Older women are more likely to participate in aerobic exercise groups<br>**D**: Active people are more likely to use the walking path<br>**D**: People with a high BMI are more likely to join lectures	**BE**: Significant increase in PA<br>**PE**: Reduce in weight gain, beneficial effects for triglyceride levels, cholesterol ratio, systolic blood pressure and glucose levels				3 years FU: Increase in PA, reduce in weight gain, cholesterol, blood pressure	
**2. 10.000 Steps Flanders**	Pilot study:<br>**Awareness**: 63,2%<br>Dissemination study:<br>**Awareness**: 35% noticed the project 33% (print media), 21% (TV), 18% (health insurance organizations), 13% (friends/ family) 22% (sale of pedometers), 9% walking circuits, 3% loan of pedometers	**Awareness:**<br>ND: age, gender, education, employment status	**BE**: Increase in daily steps, walking time, moderate, work-related and leisure time PA	**ND:** age<br>**D:** Staff members with more years of experience in PA promotion are more likely to adopt the program	**AR**: 36%<br>**ND**: number of staff members, type of organization and working context (urban-rural)	Global implementation score of 52/100 90.5 (sale or loan of pedometers), 87.5 (community events), 76.2 (flyers), 64.3 (media advertisements), 52.4 (website), 37.5 (partnerships), 35.7 (posters), 19.0 (street signs), 17.2 (personal contact)	Four years FU: the positive effects seen after one year were not maintained after 4 years	50% of the organizations continued 10.000 steps

**3. 10.000 Steps Rock-hamton**	Pilot study:<br>**Awareness**: 95%<br>**Participation:** 18,1% (pedometer)	**Awareness:**<br>**D**: Women are more aware than men<br>**Participation:**<br>**D:** Pedometer users were more likely to be female, aged 45 or older, have higher levels of education, have a BMI of 30 and higher	**BE**: No increase in PA, but prevent the fall in PA levels		Dissemination study:<br>**AR**: 95 workplaces , 13 communities	33% changed the name of the intervention		Sustained intervention
**4. Isfahan Healthy Heart Program**			**BE**: Increase in healthy diet, no drop in energy expenditure	176 public health officers	IHH worksite intervention:<br>AR: 45%	Well implemented, but only 57% of the factories could improve their food menus	Six years FU: positive effects were maintained	
**5. Agita Sao Paulo**	**Awareness**: 55,7% noticed the project of which 23,1% knew the main message	**Awareness:**<br>**ND**: socio-economic levels	**BE**: People who knew the main message are more PA and less sedentary					Sustained intervention
**6. Healthy Hawaii Initiative**			**BE**: Reduction in no leisure time PA, increased fruit and vegetables consumption<br>PE: Obesity rates remained stable				Four years FU: Increase in fruit and vegetables and decrease in sedentary people	Sustained intervention
**7. Walk the Ozarks to Wellness**			**BE**: Participants in the moderate and high dose categories were about three times more likely to meet walking guidelines					
**8. Healthwork s**			**PE**: No intervention effects on weight change		**AR**: 12% (6/52)	100% change food offerings >50% calorie smart foods and beverages in cafeteria, 0% price reduce of calorie smart foods, 66% change vending food offerings, 66% enhancing stairwell		

						attractiveness, 100% pedometer intervention, 100% scale access intervention, 100% publicity, 100% newsletters		
**9. Hartslag Limburg**	**Awareness:** 24.3%<br>**Participation:** 15%	**Awareness:**<br>Women are more familiar with nutrition and PA activities than men, older people are more familiar with PA activities and higher educated people are more familiar with nutrition and PA activities<br>**Participation:**<br>**D:** women are more likely to participate, older people are more likely to participate and **ND**: no sign. difference in education level	**PE**: Sign. changes in BMI, waist circumference, systolic blood pressure, total cholesterol en serum glucose		**AR**: 71,2%		Five years FU: positive effects on BMI, waist circumference	Sustained intervention
**10. The Well London Project**			**BE**: Increase in healthy eating, physical activity and mental health					Sustained intervention
**11. The Looma Healthy Lifestyle Program**			**BE**: Improvements in dietary intake and PA				Four years FU: decrease in fat intake and increase in PA	Sustained, now entirely ran by community members
**12. Arkansas Healthy Employee Lifestyle Program**	**Participation**: 20%	**Participation:** Most participants were female and white, but HELP was able to reach the high-risk employees (45% of participating employees were obese, 29% have overweight)	**BE**: Sign. change in eating 3 or more fruits and vegetables a day, other dietary intake did not differ significantly		**AR:** 200 county-based offices and 2 central offices			Sustained intervention
**13. Cycling connecting communiti**	**Awareness:** 13,5%	**Awareness:**<br>**D:** occasional riders are more likely to recall awareness	**BE**: Increasing use of cycling paths and minutes of cycling					Sustained

**es**		**ND**: age and gender						
**14. Concord, a great place to be active**	**Awareness**: 50,6%		**BE**: Sign. decrease in sedentary women, no sign. change in moderate and vigorous PA					
**15. NHF-NRG In balance**			**PE**: Sign. changes in skinfold thickness and waist circumference		**AR**: 9% (12/ 128)	67% places posters near the elevators and stairs, 33% formed worksite linkage boards and organized more environmental interventions (e.g. information booklets, walking routes, bike scheme)		
**16. Familias Sanas y Activas**			**PSE:** Improvements in depressed mood and anhedonia<br>**BE**: Increased use of parks and PA facilities,<br>**PE**: Improved waist circumference, blood pressure, fitness, hamstrings flexibility	30 unpaid promotores<br>**D:** all trainers were women aged between 19 and 45 years				Sustained intervention
**17. Samoan church interventio n**			**BE**: Sign. improvements in vigorous activity<br>**PE**: Sign. improvements in weight					
**18. Health-e-AME**	**Participation**: vary between 2%-100%, with a median of 18,5%	**Participation:**<br>**D**: women were more likely to participate	No sign. effects		**AR**: 66%<br>**D:** large churches are more likely to adopt the intervention<br>**ND**: geographical area, ethnicity and SES	14% (8 Steps to Fitness), 32% (walking program), 22% (praise aerobics), 18% (chair aerobics), 26% (educational activities)		Sustained intervention; 13 churches were still offering at least one component of the intervention
**19. Body and Soul**	Pilot study:<br>**Participation:** 75% attended kick-off		**BE**: Increased fruit and vegetables intake	64 church members were	**AR**: 16 churches, 25-50% adopted the program	1/8 initiated all 4 pillars (pastoral involvement,		Sustained intervention

	event, 63% attended at least one other activity<br>Dissemination study:<br>**Awareness:** 79%<br>**Participation:** 74%			volunteer advisors, of which 74% was female	**D:** Intervention agents are more likely to be higher educated and have a higher income	educational activities, church environmental changes, and peer counseling), the other initiated at least two pillar		
**20. Walk Kansas**	**Participation**: 1%	**Participation:**<br>**D**: Women were more likely to participate, older people are more likely to participate and active people were more likely to participate<br>**ND**: ethnicity	**BE**: Increase in minutes of moderate and vigorous activity		**AR**: 92%<br>**D**: Counties that adopted the intervention had higher populations		6 months FU: no decrease in PA	Sustained, 76% of the counties adopted the intervention for at least 3 years
**21. Metropolitan Transit Workers**	**Participation**: 55%		**BE**: Energy intake decreased sign, and fruit and vegetable intake increased sign., no change in PA					
**22. Merrill Lynch Company**			**PE**: Sign. improvements in BMI and systolic BP					
**23. Dow Chemical Company**			**BE**: Significant improvements in nutrition and PA				Two years FU: increase in PA	
**24. Eating for a Healthy Life**			**BE**: Significantly healthier dietary behaviors		**AR**: 60% (12/20)	5/7 implemented social activities (= healthful eating into existing activities), 3/7 healthy eating sessions, 2/7 advisory meetings, 6/7 motivational messages		Sustained
**25. Worksite Opportunities for Wellness**			**PE**: Improvements in fitness, blood pressure, and cholesterol Improvements in BMI in one worksite					
**26.**			**PSE**: Sign. increase in health-					

**Bangladesh Interventio n**			related quality of life					
**27. Elementary school personnel**	**Awareness:** 100%<br>**Participation**: 70%		**PE**: Sign. decrease in BMI					
**28. Academia da Cidade**	**Awareness:** 61.7%<br>**Participation:** 5.2%	Active people are more likely to participate in the intervention	**BE**: Moderate to high levels of leisure-time physical activity were higher among former and current intervention participants					Sustained, integrated in public health care
**29. Beijing Universities**	**Awareness**: 51,2%		**PSE**: increase in mental health<br>**BE**: Sign. decrease in PA!!! Sign. reduce in high-fat food intake			All of the intervention activities are implemented by the 6 universities		
**30. Heart at Work**			**PSE**: sign. improvements in CVD risk awareness, and self-efficacy<br>BE: No sign. effect on diet, PA, weight loss	One health care coordinator (=nurse) per workplace				
**31. Pasos adelante**			**BE**: Significant increase in moderate to vigorous walking among participants and shifts in nutritional patterns<br>**PE**: Decreases in BMI, waist and hip circumferences, total cholesterol and blood pressure	11 promotores				
**32. Wijkgezond heidswerk**	**Awareness**: 24% knew the program by name, but 70% were aware of subinterventions		**BE**: Only small impact on fruit consumption			Most key principles were carried out		Sustained (other name)
**33. Groninger Active Living**			**BE**: Sign. effect for energy expenditure for recreational sports activities, other leisure time PA and total PA					
